# Electron cryotomography of SARS-CoV-2 virions reveals cylinder-shaped particles with a double layer RNP assembly

**DOI:** 10.1038/s42003-022-04183-1

**Published:** 2022-11-10

**Authors:** Lesley J. Calder, Thomas Calcraft, Saira Hussain, Ruth Harvey, Peter B. Rosenthal

**Affiliations:** 1 Structural Biology of Cells and Viruses Laboratory, The Francis Crick Institute, NW1 1AT London, United Kingdom; 2 Worldwide Influenza Centre, The Francis Crick Institute, NW1 1AT London, UK; 3 RNA Virus Replication Laboratory, The Francis Crick Institute, NW1 1AT London, UK

**Keywords:** Virology, Cryoelectron tomography

## Abstract

SARS-CoV-2 is a lipid-enveloped Betacoronavirus and cause of the Covid-19 pandemic. To study the three-dimensional architecture of the virus, we perform electron cryotomography (cryo-ET) on SARS-Cov-2 virions and three variants revealing particles of regular cylindrical morphology. The ribonucleoprotein particles packaging the genome in the virion interior form a dense, double layer assembly with a cylindrical shape related to the overall particle morphology. This organisation suggests structural interactions important to virus assembly.

## Abstract (web summary)

Electron cryotomography expands our knowledge of SARS-CoV-2 virus architecture and reveals a distinct and uniform morphology for which interactions between RNP subunits within the nucleocapsid and between the nucleocapsid and the membrane are likely to be important

## Introduction

SARS-Cov-2, the causative agent of Covid-19, has been the object of intense investigation, including structural studies by cryo-EM of individual component proteins at high-resolution, as well as cryotomography of viruses^1–3^ and infected cells^4,5^. Cryotomography of frozen-hydrated virus particles enables the visualisation of particle exterior and interior in three dimensions and thus is an important method for understanding the three-dimensional architecture of pleomorphic lipid-enveloped viruses. These previous studies have described SARS-CoV-2 virions as spherical or ellipsoidal and in addition have mainly visualised the spike protein (S) in pre-fusion and post-fusion states on the virus surface and ribonucleoprotein assemblies in the virus interior. Other virus structural components such as the membrane protein (M), the envelope protein (E) and additional non-structural proteins may play important roles in virus structure and assembly.

Because a number of fundamental questions remain about the virus architecture, such as how virion components interact in three-dimensions to assemble particles and how the large positive strand genome is packaged, we apply cryotomography to study the architecture of virions of the original Wuhan strain of SARS-CoV-2 and three variants that arose during the first year of the pandemic. The tomograms show the particles are of a uniform cylindrical shape with spike proteins distributed over the whole envelope. The interior of the particle reveals an organised RNP assembly with implications for virus assembly.

## Results and Discussion

**Fig. 1:**
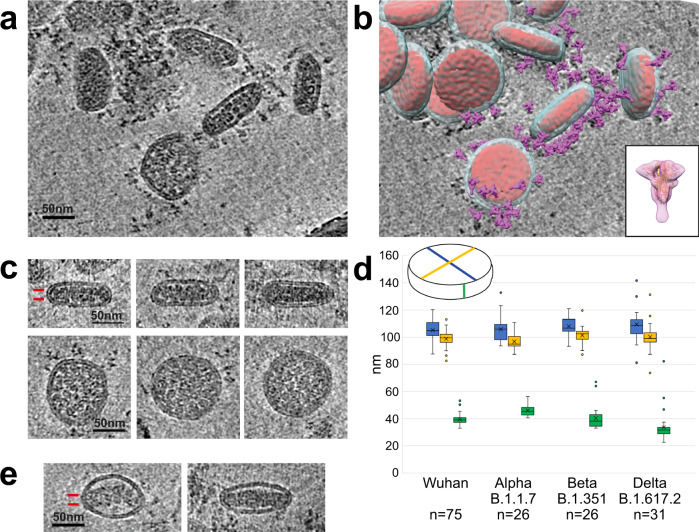
Electron cryotomography of SARS-CoV-2 virions. **a** Tomogram section of Wuhan virions in cross-section showing surface spike proteins surrounding the viral membrane bilayer and the nucleocapsid in the virus interior. **b** Same field of view with models for the virus membrane (pale blue), nucleocapsid (red), and the spike glycoprotein (purple) overlaid. The glycoprotein map (inset) was obtained by subtomogram averaging of spikes and fitted with a pre-fusion S model (pdbid 6zge). **c** Gallery shows cross-sections through narrow side views and circular top views of Wuhan virions. The side view shows two layers of density for RNP subunits in the nucleocapsid assembly (indicated by two red lines), which is separated from the top and bottom membrane bilayer. The circular top views show dense RNP subunits that extend to the bilayer. **d** Virion dimensions for 4 variants (Wuhan *n* = 75, Alpha *n* = 26, Beta *n* = 26, Delta *n* = 31). Box and whisker plot of the measurements of 3 orthogonal dimensions as shown in inset: circular view major axis (blue), circular view minor axis (yellow), side view (green). The boxes represent the interquartile range of measurements, line across box = median, x inside box = mean (see Supplementary Table 1 for values), whiskers extend to minimum and maximum measurements, circles = outliers. **e** Wuhan virions of aberrant ellipsoidal morphology show a two layer nucleocapsid, which abuts the membrane at the highly-curved sides while remaining separated from the membrane bilayers above and below.

**Fig. 2:**
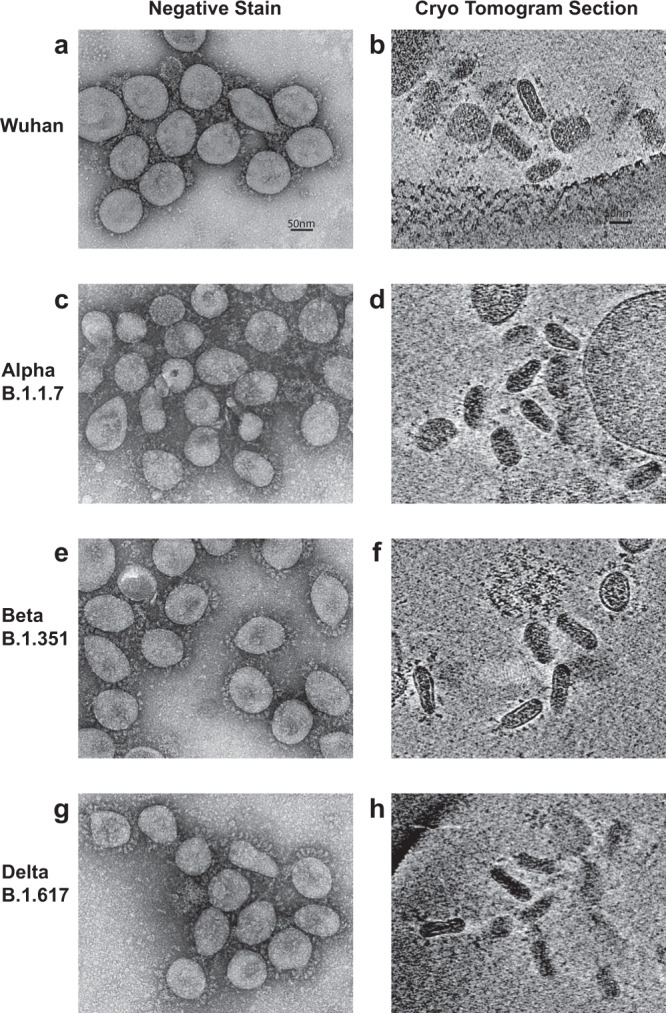
Particle fields for Wuhan strain virus and three variants as seen in negative stain micrographs on a carbon support and in cryo-ET sections. Wuhan strain in negative stain (**a**) and cryo-ET (**b**). Alpha strain (B.1.1.7) in negative stain (**c**) and cryo-ET (**d**). Beta strain (B.1.351) in negative stain (**e**) and cryo-ET (**f**). Delta strain (B.1.617) in negative stain (**g**) and cryo-ET (**h**). The negative stain fields show predominantly circular views of virions, while the tomogram sections show circular, elliptical side, and oblique views. All four variants show similar virion profiles decorated with Spike proteins.

We recorded tilt-series and reconstructed cryotomograms of frozen-hydrated SARS-CoV-2 virions from the original Wuhan strain and Alpha (B.1.1.7), Beta (B.1.351) and Delta (B.1.617) variants. The tomograms show that the virions are predominantly of a single and uniform morphology which is an extremely flat cylindrical shape. A slice through a tomogram of Wuhan strain virions shown in Fig. 1a, b is typical of three independent preparations of the virus. The virions are randomly oriented within the ice layer and therefore a variety of oblique sections through the virions are observed in each slice of the tomogram, including circular and elliptical profiles, similar to those previously described for SARS-CoV-2^3^ and other coronaviruses^6–8^. A gallery of Wuhan virion cross-sections is shown in Fig. 1c and Supplementary Fig. 1a,b,c. Each virion has a cross section corresponding to a circular (‘’top view”) and in orthogonal directions a cross section corresponding to a narrow (‘’side view”), as shown in Supplementary Fig. 2. The observed shape of the virions is not the result of compression during sample preparation in thin frozen-hydrated films which could potentially flatten spherical particles because top and side views occur in any orientation with respect to the specimen plane in our tomograms. In contrast, the two-dimensional projections of virions absorbed to a carbon support film and observed by negative stain show predominantly circular views by comparison to diverse views in images of frozen-hydrated specimens of the same virus preparations (Fig. 2). This is consistent with a preferential arrangement of the flat cylindrical virions on the supporting carbon film such that the largest surface area is in contact with the support film.

We have measured the three principal axis dimensions as shown in Fig. 1d. The cylinder cross section is slightly elliptical, and the cylinder height (Wuhan average = 39 nm) is less than half that of the average diameter (Wuhan average = 102 nm). Similar shapes and dimensions were observed and measured from cryotomograms for the Alpha, Beta and Delta variants (Fig. 1d, Fig. 2, Supplementary Table 1, and Supplementary Fig. 1).

We observe spike proteins protruding from the viral membrane with a highly flexible linkage (Fig. 1a and Supplementary Fig. 1c) as previously described for SARS-CoV-2^1–3^ and which we also observed for the variants (Fig. 2 and Supplementary Fig. 1). We obtained a low-resolution subtomogram average of Wuhan spikes in agreement with models^9–11^ for the prefusion conformation (Fig. 1b**, inset**). We placed copies of the pre-fusion spike map at their locations in the virion tomograms (Fig. 1b) where they are widely spaced over the whole virus membrane as observed in previous tomographic studies^1–3^. 3D classification of the spike particles show pre-fusion forms including closed spikes and spikes with one receptor binding domain (RBD) up (Supplementary Fig. 3, Table 1). We also see rod-like densities on the membrane consistent with a “postfusion” conformation^1–3,12–14^ of the glycoprotein (Supplementary Fig. 1c). While the number of spikes per particle shows some variation, the cylindrical morphology appears to be independent of their number and of whether pre-fusion or, as in rare cases, post-fusion spikes predominate (see examples in Supplementary Fig. 1c).

**Table 1:** Cryo-EM data collection, refinement and validation statistics.

	Spike, closed (EMDB-15181)	Spike, 1-RBD-up (EMDB-15185)	Whole virion (EMDB-15183)
**Data collection and processing**			
Magnification	64,000	64,000	64,000
Voltage (kV)	300	300	300
Electron exposure (e^–^/Å^2^)	98.4	98.4	98.4
Defocus range (μm)	−2.5 to −5 µm	−2.5 to −5 µm	−2.5 to −5 µm
Pixel size (Å)	4.44	4.44	8.88
Symmetry imposed	C3	C1	C1
Initial particle images (no.)	4418	4418	268
Final particle images (no.)	321	474	268
Map resolution (Å) FSC threshold = 0.143	29.6	27.8	44.4

**Fig. 3:**
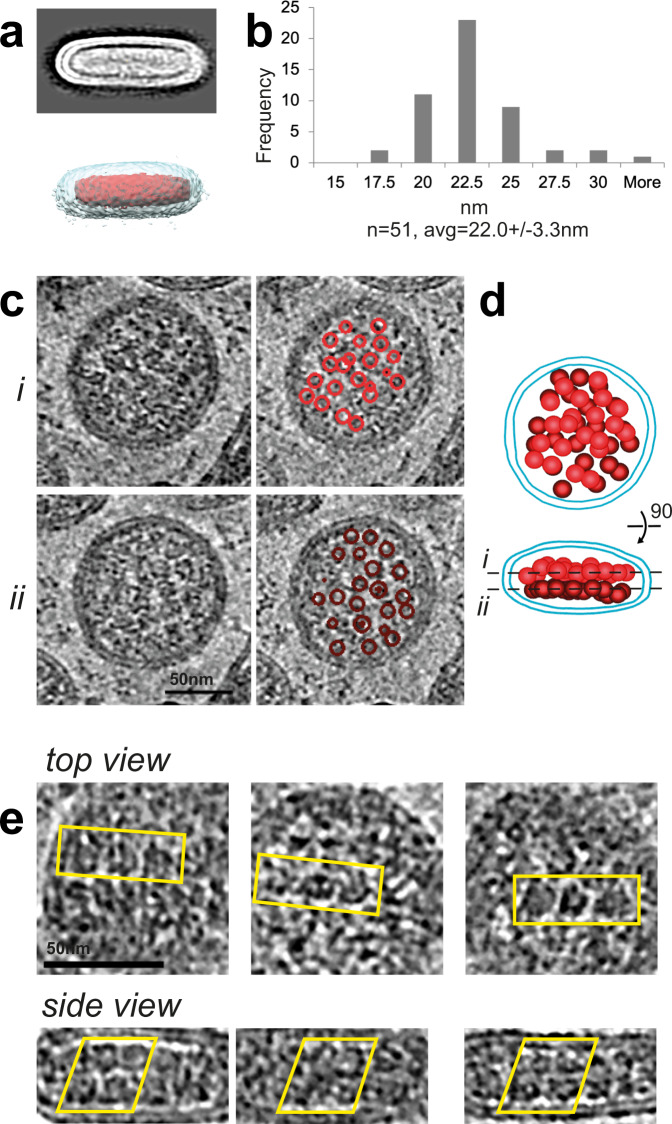
RNP features in Wuhan SARS-CoV-2 virions. **a** Average map (top) of *n* = 268 virions showing membrane bilayer and internal nucleocapsid double layer and map segmentation (bottom) to indicate envelope (pale blue) and nucleocapsid (red). **b** Histogram of measured height of two layer cylindrical nucleocapsid (*n* = 51 nucleocapsids, avg=22, s.d. 3 nm). **c** Two tomogram sections through the same virion 12 nm apart (i, ii) and the same sections with spherical RNP subunits identified by red circles (*i*: bright red, *ii*: dark red). Different subunits are labelled in each section. **d** Model of virion in c showing bilayer in cyan and RNP subunits as 13 nm red spheres with sections in c indicated by dotted lines. 45 RNP subunits are shown in this virion which is at the top end of size range. **e** High magnification gallery of virions showing top circular and side views identifying arrays of RNP subunits (outlined in yellow boxes). The side views show a staggered and inter-digitated packing of RNP subunits.

Because of the relative uniformity of the cylindrical virus particles, we were able to calculate average maps from sub-volumes containing whole Wuhan virions, showing the membrane bilayer and two layers comprising the nucleocapsid assembly (Fig. 3a). Principal component analysis (PCA) shows a small variability in the cylindrical height of the virions (Supplementary Fig. 4).

The virion interior contains a dense assembly of ribonucleoproteins (RNPs) that package the genome to form the nucleocapsid. The nucleocapsid has a cylindrical shape that matches the particle geometry. By comparison, particles described at budding sites in the ERGIC compartment of the ER have less compact interiors^4^. The compaction observed here is reminiscent of particle maturation observed in imaging studies of transmissible gastroenteritis coronavirus^15^.

The RNPs are arranged in a two-layer cylindrical assembly of height 22 nm (Wuhan average) as shown in Fig. 1c, Fig. 3 and Supplementary Fig. S1b. Individual RNP subunits are more easily discerned in virions with dimensions at the high end of the measured range, as illustrated in Fig. 3 c,d. In the circular cross-section, some subunits have a diameter (15 nm) and appearance similar to RNP models obtained by subtomogram averaging of RNPs in virus^3^, and are also similar to particles assembled in vitro from N (nucleocapsid) protein and RNA^16^. The side cross-section shows compact packing of RNP subunits within a layer and typically staggered packing between layers (Fig. 3e). We make similar observations about the RNPs of the other variants (Supplementary Fig. 1 d,e,f). By comparison the spherical or elliptical particles shown in previous studies^1–3^ typically have less compact interiors in which the RNPs are more separated.

In the circular top-view cross-section the nucleocapsid contacts the membrane around its circumference, suggesting association of the RNPs with a membrane component, such as the membrane protein (M). In the side-view cross-section, the nucleocapsid is only contiguous with the membrane at the highly curved ends (Fig. 1c), and there is a gap between the nucleocapsid and the flat top and bottom membranes, which appears devoid of an abutting protein layer (Fig. 1c, Supplementary Fig. S1). Interaction with the M protein may therefore be concentrated at points of high membrane curvature consistent with observations for SARS-CoV^8^. A membrane association of the nucleocapsid cylindrical circumference is also apparent in virions with more ellipsoidal shapes (Fig. 1e). These aberrant particles are rare, about 1% of the total Wuhan strain population, but offer insight into how the nucleocapsid associates with the viral membrane. The double-layer RNP assembly, contacting the viral membrane at the highly curved ends and not at the flat top and bottom membranes, is consistent among all the variants (Supplementary Fig. S1 d,e,f).

RNP-RNP interactions may themselves form a cylinder-shaped condensate that interacts with a membrane component and shapes the surrounding membrane. Alternatively, membrane-associated proteins that are organised by interaction with the RNP assembly may also play a role in creating membrane curvature and determine particle morphology. These association properties are consistent with biochemical characterisation of the nucleoprotein, N, which has been observed to interact and form condensates with RNA or M protein, potentially in annular structures, and to interact with membranes via the M protein^17^.

Our observations of a distinct and uniform morphology of the SARS-CoV-2 virion and its organised interior indicate the importance of interactions between RNP subunits within the nucleocapsid, and between the nucleocapsid and membrane components. During the assembly of new virus particles these interactions may determine the overall shape of the virion and may also play roles in particle disassembly following cell entry by membrane fusion.

## Methods

### SARS CoV-2 virus seed stock production

Four different viral strains were obtained as follows:

Wuhan (hCoV-19/England/02/2020), (GISAID EpiCov™ accession EPI_ISL_407073) from The Respiratory Virus Unit, Public Health England (PHE), UK.

Alpha variant B.1.1.7 (hCoV-19/England/204690005/2020) from PHE through Professor Wendy Barclay, Imperial College, London, UK.

Beta variant B.1.351 (501Y.V2.HV001) from the African Health Institute, Durban, South Africa.

Delta variant B.1.617.2 (GISAID accession EPI_ISL1731019) from Professor Wendy Barclay, through Genotype-to-Phenotype National Virology Consortium.

All viruses were grown at the World Influenza Centre, Francis Crick Institute, London, UK under Biosafety level 3 conditions in Vero V1 cells, provided by Professor Steve Goodbourne, St George’s Hospital, University of London, UK and maintained in Dulbecco’s Modified Eagle Medium (DMEM) Gibco™ 41965039, with 100 U/ml penicillin, 100 μg/ml streptomycin (Pen-Strep) and 10% (v/v) heat-inactivated fetal calf serum (FCS).

### Viral growth and purification for EM

Vero V1 cells were infected at low multiplicity of infection (MOI) of 0.0001PFU per cell with viral seed stock and incubated as above for 4-5 days until about 90% cytopathic effect (CPE) was observed before the supernatant was harvested. The low MOI and long incubation time was chosen to ensure a multiple cycle infection thus maximising the proportion of infective to defective virus particles that are produced with low cycles of infection. The length of incubation time was experimentally identified to maximise the virus yield. The supernatant was harvested and clarified twice by centrifugation at 3180 g for 30 mins at 4 °C in an Allegra 12-R centrifuge, SX4750 rotor. Virus was pelleted from the supernatant through 30% (w/v) sucrose in 10 mM MOPS, 150 mM NaCl, 1 mM EDTA, pH 6.8 (MES buffer), spun at 111,063 g for 90 mins at 4 °C in an Optima XPN-90 Beckman ultracentrifuge, SW32Ti rotor. Viral pellets were resuspended overnight in MES buffer at 4 °C and then formaldehyde added to a final concentration of 4% (v/v), incubated at 20 °C for 20 mins and then stored overnight at 4 °C.

### Negative stain electron microscopy

See Supplementary Table 1 for numbers of viral preparations analysed.

All virus preparations were screened by negative stain EM.

2 μl drops of sample were absorbed to a carbon-coated 400 mesh copper grid (TAAB). After 30 s the grid was floated onto water, sample side in contact with the water for 30 s, then transferred to 1% sodium silicotungstate pH 7.5 (Agar Scientific) for 30secs. The grid was air dried, viewed and imaged at a pixel size of 7 Å with a Technai Spirit TEM (FEI) operated at 120 kV, with an Eagle 4 K detector (FEI).

### Cryo-EM grid preparation

Virus samples were mixed with 10 nm Protein A gold (BBI Solutions) in a ratio of 4:1 by volume. 3.5 μl drops were applied to air glow-discharged 200 mesh copper Quantifoil R2/2 grids and plunge frozen in liquid ethane using a MkIV Vitrobot (Thermo Fisher) operated at 90% humidity, 4 °C.

### Electron cryotomography (Cryo-ET)

See Supplementary Table 1 for number of tomograms collected for each variant and with which microscope.

The Talos microscope, fitted with a Falcon 3 camera, was operated at 200 kV. Tomograms were collected using the Thermo Fisher Tomography 5 software in linear mode, using a dose-symmetric scheme at 3° intervals to + /− 57° at a pixel size of 2.58 Å, and total dose of 94e^-^/Å^2^.

The Titan Krios microscope is fitted with a Gatan GIF Quantum energy filter operated in zero-loss mode with a 20 eV slit width, and a K2 Summit detector (Gatan) operated in counting mode. Dose-symmetric tilt series were aquired using the Thermo Fisher Tomography 5 software at 3° intervals to + /−60°, using a defocus range of −2.5 to -5um, at a pixel size if 2.22 Å and 4 frames per tilt image. The total tomogram dose was 98e^-^/Å^2^.

### Tomogram generation

Talos-acquired tilt series were fiducial aligned with the IMOD package^18^ and reconstructed with SIRT, 5 iterations. Resulting tomograms were binned 4x and filtered with 10 iterations of Nonlinear Anisotropic Diffusion.

Movie frames of Krios acquired tilt series were motion corrected, dose-weighted and fiducial-aligned using the IMOD package. The contrast transfer function was estimated with CTFFIND4^19^, tomograms were CTF corrected by phase flipping and reconstructed with novaCTF^20^, producing a weighted back-projection tomogram and a SIRT-like filtered tomogram. For visual analysis the SIRT-like filtered tomograms were binned 4x and subjected to 20 iterations of Nonlinear Anisotropic Diffusion filtering using the Bsoft^21^ program bnad with default parameters (λ = 0.1).

### Subtomogram averaging

268 whole virions from 20 tomograms were picked manually from the 4-fold binned SIRT/NAD-filtered tomograms using EMAN 2.91^22^. Particles were then extracted from the unbinned, CTF-corrected WBP tomograms with 4-fold downsampling (box size 1598 Å, 8.88 Å/pixel sampling) using Relion 3.1^23^. Reference-free alignment of all subtomograms produced an initial map which was used as a reference for further 3D classification and alignment. A loose, soft mask around the outer surface of the virion envelope was used during further alignment, classification and postprocessing. The average map of 2-layered virions was estimated to have a resolution of 44 Å at the FSC = 0.143 cutoff by Relion postprocessing. After the alignment of all subtomograms, 3D classification into 5 classes without alignment was then carried out to examine the varying morphologies of the virions.

For PCA analysis, the virion subtomograms were re-imported into EMAN 2.91^22^, aligned against the Relion map and analysed using PCA-based classification, starting from 3 initial basis vectors and requesting 3 output classes.

Spike particles were picked manually from the 4-fold binned SIRT/NAD-filtered tomograms using IMOD. 4418 particles from 251 virions in 18 tomograms were then extracted from the full-size WBP tomograms with 2-fold downsampling (box size 444 Å, 4.44 Å/pixel sampling) for subtomogram averaging in Relion 3.1. Reference-free initial model generation in C1 produced a map with clear 3-fold features, which was symmetrised and used as a reference for further refinement with C3 symmetry applied and with a loose mask around the ectodomain. Upon convergence of refinement, the particles were reclassified with relaxation of C3 symmetry, revealing a class with the 1-RBD-up conformation. The classes were separated and the closed conformation particles were finally refined with C3 symmetry, while the 1-RBD-up particles were refined without symmetry. The final resolutions were estimated as 30 Å (closed conformation) and 28 Å (1-RBD-up conformation) at the FSC = 0.143 cutoff by Relion postprocessing.

The subtomogram averaging maps were backplotted into the frame of reference of the original tomogram using in-house scripts (available from the authors). Spike particles were visually inspected and removed if they were misaligned (i.e. had a relative tilt of over 90° with respect to the normal of the viral envelope) or duplicate particles which converged to the same position during alignment.

### Statistics and reproducibility

Number of biological replicates and measurements made are given in Supplementary Table 1.

### Reporting Summary

Further information on research design is available in the Nature Research Reporting Summary linked to this article.

## Supplementary information

Supplementary Information

Description of Additional Supplementary Data

Supplementary Data 1

Reporting Summary-New

## Data Availability

Tomograms and subtomogram reconstructions have been deposited in the Electron Microscopy Data Bank, http://www.ebi.ac.uk/pdbe/emdb/ (Accession Nos. EMD-15181, EMD-15182, EMD-15183, EMD-15185). Image tilt-series have been deposited in the Electron Microscopy Public Image Archive (EMPIAR) with accession code EMPIAR-11070. Source data and calculations for Fig. 1d are provided as Supplementary Data 1.
