# Supplementary material for: Electron cryotomography of SARS-CoV-2 virions reveals cylinder-shaped particles with a double layer RNP assembly
Source: Commun Biol. 2022 Nov 10;5:1210. doi: 10.1038/s42003-022-04183-1 (PMC9648435; doi:10.1038/s42003-022-04183-1)
Supplement: Supplementary file 1 — Supplementary Information [file 42003_2022_4183_MOESM1_ESM.pdf]

Supplementary Information

Electron cryotomography of SARS-CoV-2 virions reveals cylinder-shaped particles with a double layer RNP assembly

Lesley J. Calder, Thomas Calcraft, Saira Hussain, Ruth Harvey and Peter B. Rosenthal

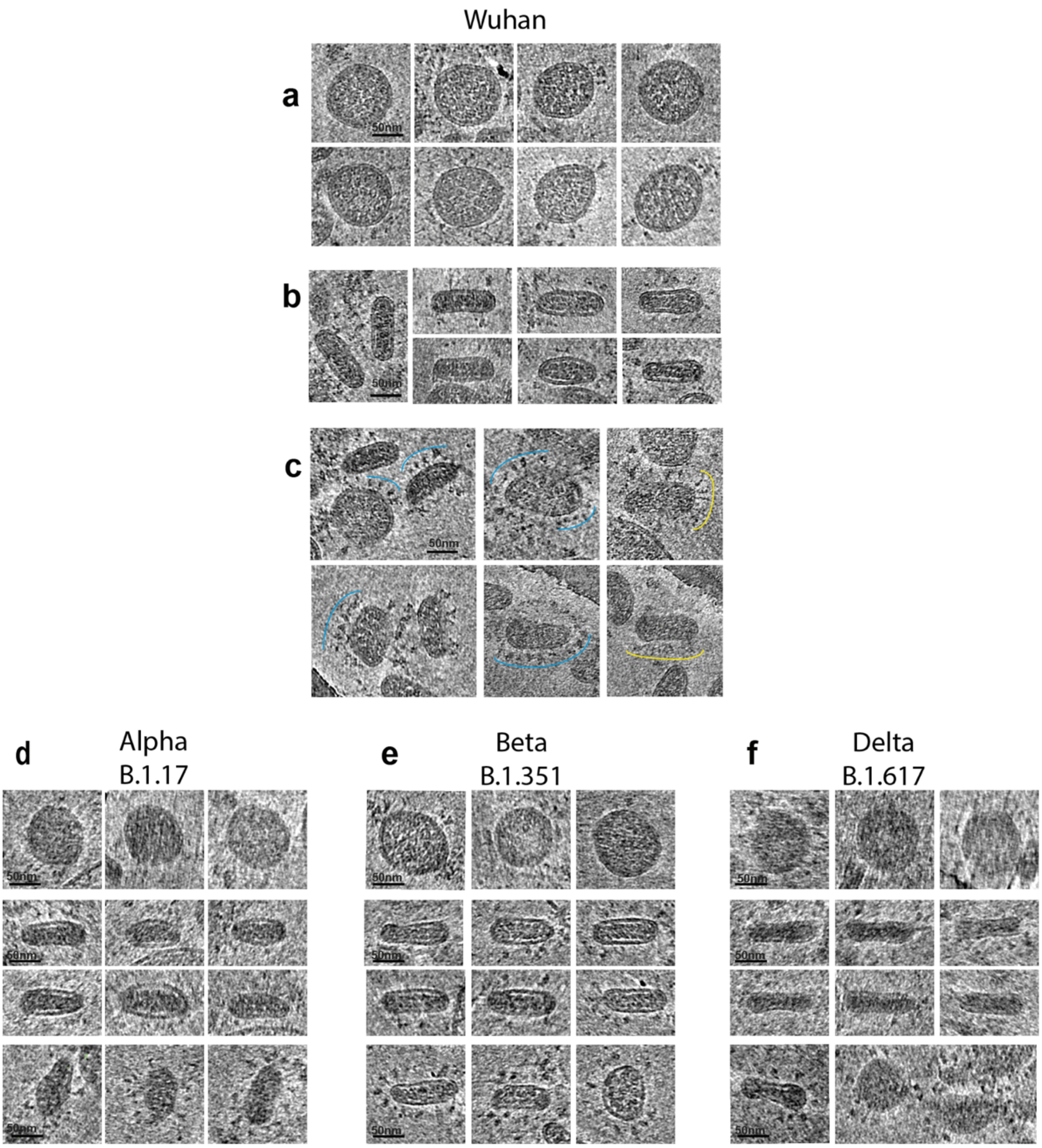

Supplementary Figure 1. Gallery of SARS-CoV-2 particles from cryo-tomograms

a) Gallery of Wuhan virions in circular top view. **b)** Gallery of virions in narrow side view. **c)** Gallery of virions, some in oblique cross section, showing surface spike proteins in pre-fusion conformation (blue arcs) and post-fusion conformation (yellow arcs). **d)** Alpha variant B1.17 virions **e)** Beta variant B.1.351 virions, and **f)** Delta variant B1.617 virions. Magnification is the same for all panels.

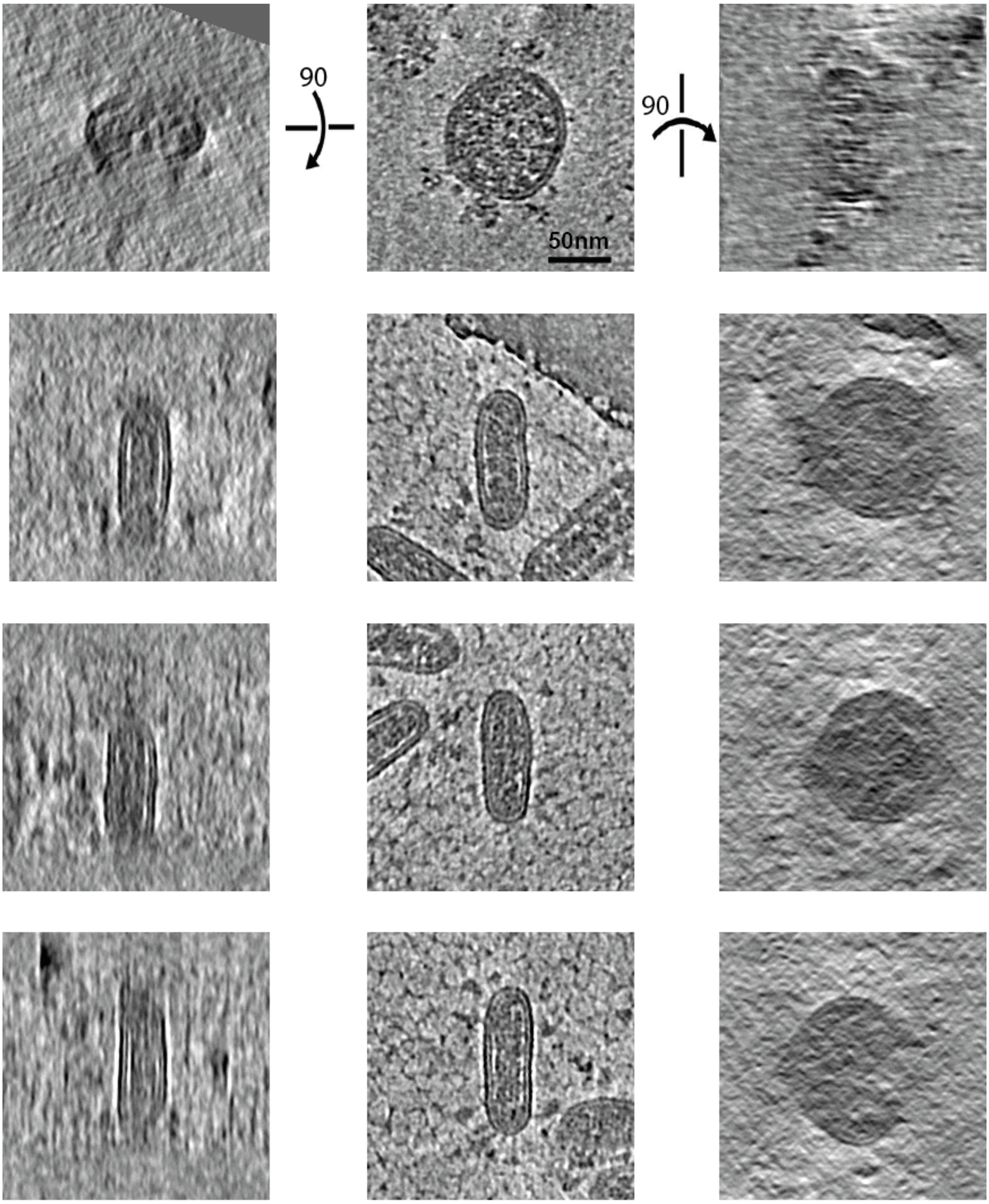

Supplementary Figure 2. Tomogram analysis of Wuhan virions.
Tomogram sections showing four virions along three orthogonal directions as labelled in Figure 1d. Centre panels are z sections of the tomogram, left and right correspond to x and y sections of the tomogram. Note: characteristic blurring is due to the missing wedge. Magnification is the same for all panels.

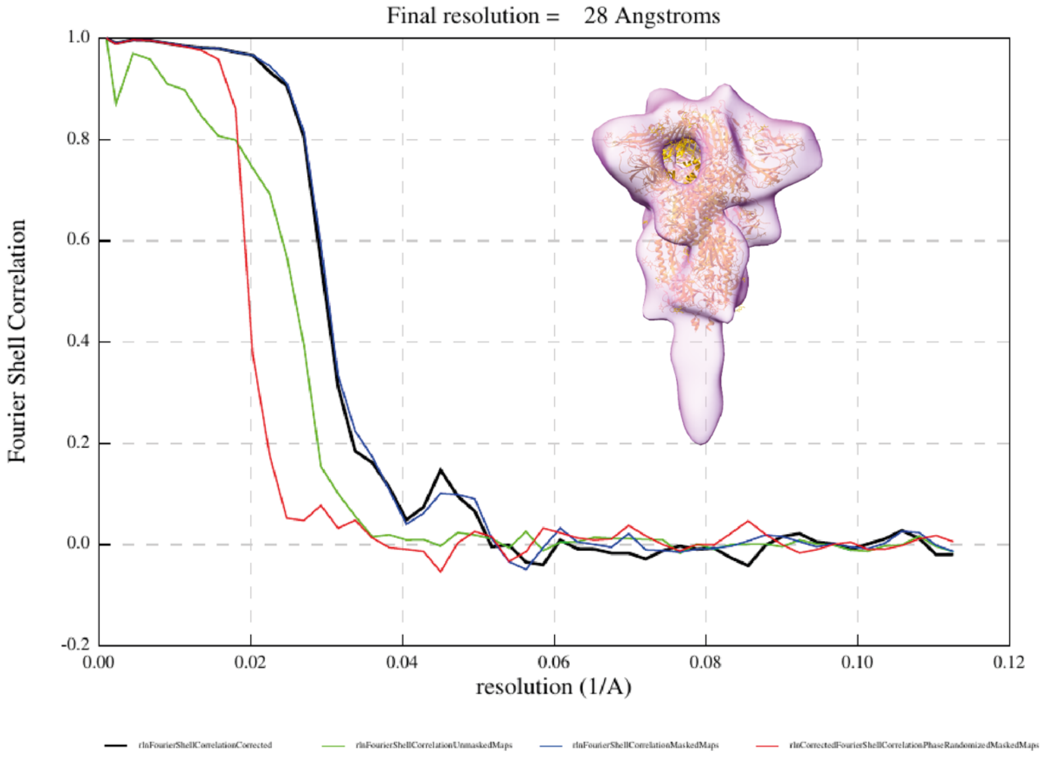

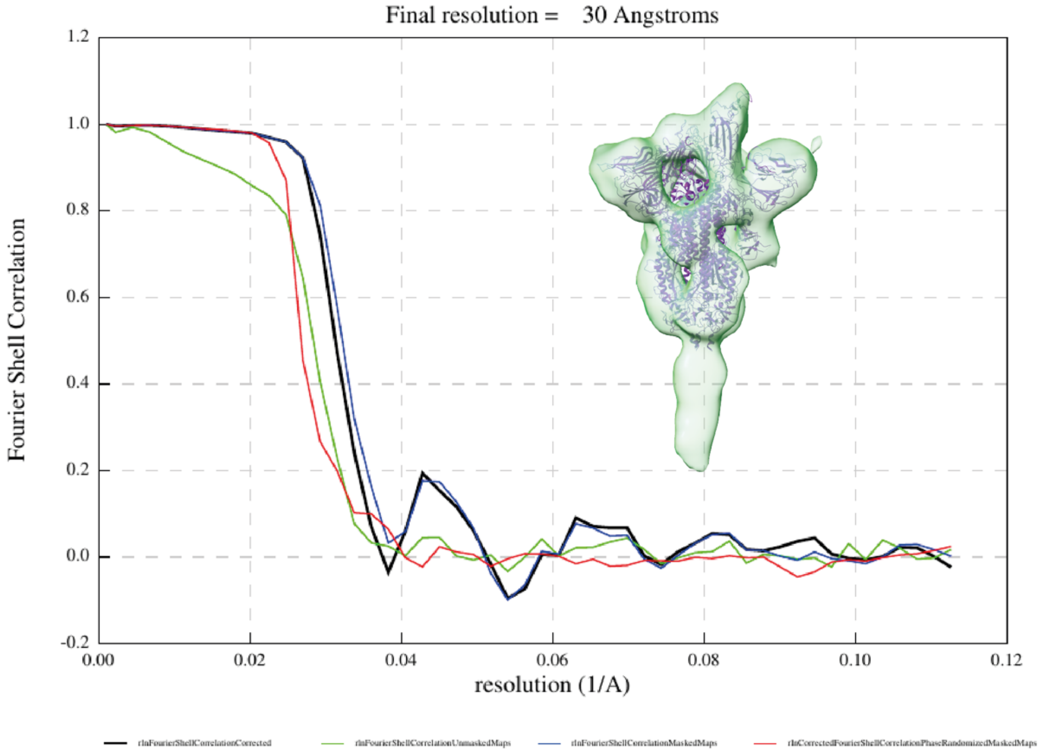

Supplementary Figure 3. 3D reconstruction of spike protein from Wuhan strain virions.
a) Closed form of the spike. FSC indicates 28 Å resolution map (0.143 threshold). Map is fit by spike model 6zge. **b)** Open form of the spike (one RBD erect). FSC indicates 30 Å map (0.143 threshold). Map is fit by model 6zgg. See also Table 1.

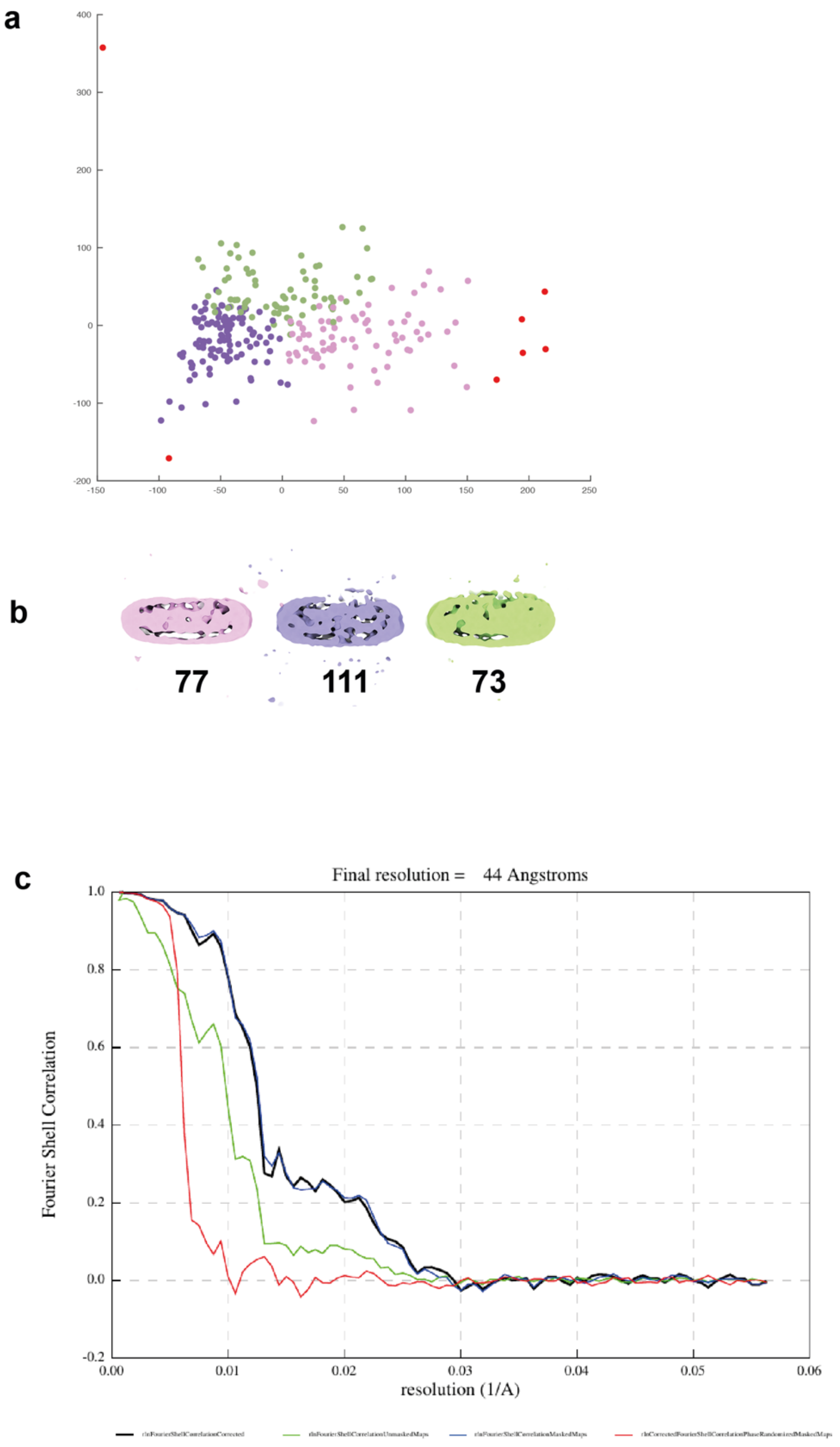

Supplementary Figure 4. PCA of Wuhan Virions and FSC analysis of virion average

a) Principal component analysis (PCA) of 3D virion structure as descried in Methods showing a continuum of particle shapes. Three classes containing 77, 111, and 73 particles out of 268 have points coloured as in b. Seven outliers in the analysis are red points. **b)** Virions are highly uniform but are a continuum between the narrowest cylindrical (pink) and the most ellipsoidal (green) class. **c**) FSC plot for virion average shown in Figure 3a indicates 44 Å (0.143 threshold).

Supplementary Table 1: Summary of SARS-CoV2 virus particle analysis

**Variant:**	**Wuhan**			**Alpha**	**Beta**	**Delta**	
Number of virus preparations analysed	3			1	1	2	
	Prep1	Prep2	Prep3			Prep1	Prep2
Number of cryo tilt series acquired	34 Krios	15 Talos	12 Talos	7 Talos	13 Talos	13 Talos	14 Talos
Number of tomograms aligned	21	3	3	3	3	3	3
Number of virions measured	75	30	12	26	26	31	23
Circular Major Axis avg /nm	105 (stdv 6.7)	108.9 (stdv 7.5)	103.5 (stdv 9.2)	105.9 (stdv 9.0)	108.0 (stdv 7.0)	109.4 (stdv 11.2)	107.0 (stdv 9.7)
Circular Minor Axis avg /nm	98.8 (stdv 5.7)	100.5 (stdv 5.3)	96.3 (stdv 9.3)	96.8 (stdv 5.2)	101.4 (stdv 6.5)	100.2 (stdv 9.6)	97.8 (stdv 8.5)
Cylinder Height avg /nm	39.4 (stdv 3.7)	41.5 (stdv 6.8)	37.5 (stdv 4.8)	46.1 (stdv 4.5)	40.3 (stdv 8.5)	33.6 (stdv 10.9)	33.0 (stdv 4.9)
