# Supplementary material for: Electron cryotomography of SARS-CoV-2 virions reveals cylinder-shaped particles with a double layer RNP assembly
Source: Commun Biol. 2022 Nov 10;5:1210. doi: 10.1038/s42003-022-04183-1 (PMC9648435; doi:10.1038/s42003-022-04183-1)
Supplement: Supplementary file 2 — Description of Additional Supplementary Data [file 42003_2022_4183_MOESM2_ESM.docx]

**Description of Additional Supplementary Files**

**File name:** Supplementary Data 1

**Description:** Source data and calculations for Figure 1d.
