# Supplementary material for: Electron cryotomography of SARS-CoV-2 virions reveals cylinder-shaped particles with a double layer RNP assembly
Source: Commun Biol. 2022 Nov 10;5:1210. doi: 10.1038/s42003-022-04183-1 (PMC9648435; doi:10.1038/s42003-022-04183-1)
Supplement: Supplementary file 4 — Reporting Summary-New [file 42003_2022_4183_MOESM4_ESM.pdf]

nature portfolio

Corresponding author(s): Peter Rosenthal

Last updated by author(s): September 25, 2022

Reporting Summary

Nature Portfolio wishes to improve the reproducibility of the work that we publish. This form provides structure for consistency and transparency in reporting. For further information on Nature Portfolio policies, see our Editorial Policies and the Editorial Policy Checklist.

Statistics

For all statistical analyses, confirm that the following items are present in the figure legend, table legend, main text, or Methods section.

n/a	Confirmed	
☐	☒	The exact sample size (*n*) for each experimental group/condition, given as a discrete number and unit of measurement
☐	☒	A statement on whether measurements were taken from distinct samples or whether the same sample was measured repeatedly
☒	☐	The statistical test(s) used AND whether they are one- or two-sided<br>*Only common tests should be described solely by name; describe more complex techniques in the Methods section.*
☒	☐	A description of all covariates tested
☒	☐	A description of any assumptions or corrections, such as tests of normality and adjustment for multiple comparisons
☐	☒	A full description of the statistical parameters including central tendency (e.g. means) or other basic estimates (e.g. regression coefficient) AND variation (e.g. standard deviation) or associated estimates of uncertainty (e.g. confidence intervals)
☒	☐	For null hypothesis testing, the test statistic (e.g. *F*, *t*, *r*) with confidence intervals, effect sizes, degrees of freedom and *P* value noted<br>*Give P values as exact values whenever suitable.*
☒	☐	For Bayesian analysis, information on the choice of priors and Markov chain Monte Carlo settings
☒	☐	For hierarchical and complex designs, identification of the appropriate level for tests and full reporting of outcomes
☒	☐	Estimates of effect sizes (e.g. Cohen's *d*, Pearson's *r*), indicating how they were calculated

Our web collection on statistics for biologists contains articles on many of the points above.

Software and code

Policy information about availability of computer code

Data collection	CryoEM data collected using Thermo Scientific Tomography 5
Data analysis	CryoEM data processed using following packages: novaCTF, Relion3.1, CTFfind4 v4.1.5, IMOD 4.10, Bsoft 2.0, EMAN2.91

For manuscripts utilizing custom algorithms or software that are central to the research but not yet described in published literature, software must be made available to editors and reviewers. We strongly encourage code deposition in a community repository (e.g. GitHub). See the Nature Portfolio guidelines for submitting code & software for further information.

Data

Policy information about availability of data

All manuscripts must include a data availability statement. This statement should provide the following information, where applicable:

- Accession codes, unique identifiers, or web links for publicly available datasets
- A description of any restrictions on data availability
- For clinical datasets or third party data, please ensure that the statement adheres to our policy

Tilt-series supporting tomogram calculation are deposited in the EMPIAR database under code 11070.

Field-specific reporting

Please select the one below that is the best fit for your research. If you are not sure, read the appropriate sections before making your selection.

☒ Life sciences ☐ Behavioural & social sciences ☐ Ecological, evolutionary & environmental sciences

For a reference copy of the document with all sections, see nature.com/documents/nr-reporting-summary-flat.pdf

Life sciences study design

All studies must disclose on these points even when the disclosure is negative.

Sample size	Sample size was determined by the number of virions of each type visible in the microscope under the described experimental condition.
Data exclusions	All recorded cryotomography tilt series were analysed unless rejected based on too few fiducials for alignment, or containing fewer than 3 viral particles. CryoEM subtomograms were included and excluded from averages within the image processing workflow standard image processing techniques such as 2D and 3D classifications. Backplotting of subtomograms to original locations in tomograms was performed automatically based on subtomogram alignment parameters and validated or rejected by visual inspection.
Replication	3 separate virus preparations were grown and analysed by cryotomography for the Wuhan strain, 2 for the Delta variant, and 1 each for the Alpha and Beta variants. One of the Wuhan preparations was studied by for subtomogram averaging, where maps were determined and assessed using independent half datasets, according to standard procedures in cryoEM.
Randomization	Not applicable to this study, as samples were not assigned to experimental groups and data were collected and processed according to standard techniques for cryoEM.
Blinding	Not applicable to this study, as there was no experimental group allocation in data collection and analysis.

Reporting for specific materials, systems and methods

We require information from authors about some types of materials, experimental systems and methods used in many studies. Here, indicate whether each material, system or method listed is relevant to your study. If you are not sure if a list item applies to your research, read the appropriate section before selecting a response.

Materials & experimental systems

n/a	Involved in the study	
☒	☐	Antibodies
☐	☒	Eukaryotic cell lines
☒	☐	Palaeontology and archaeology
☒	☐	Animals and other organisms
☒	☐	Human research participants
☒	☐	Clinical data
☒	☐	Dual use research of concern

Methods

n/a	Involved in the study	
☒	☐	ChIP-seq
☒	☐	Flow cytometry
☒	☐	MRI-based neuroimaging

Eukaryotic cell lines

Policy information about cell lines

Cell line source(s)	Vero V1 cells were obtained from Prof. S. Goodbourne, St George's Hospital, University of London, UK.
Authentication	The cell line was not authenticated.
Mycoplasma contamination	The cell line tested negative for mycoplasma.
Commonly misidentified lines (See ICLAC register)	*Name any commonly misidentified cell lines used in the study and provide a rationale for their use.*
